
JOURNAL INFORMATION
==============================
NLM Title Abbreviation: ScientificWorldJournal
Journal ID: ScientificWorldJournal
NLM Journal ID: 101131163
Journal ID: TSWJ

The Scientific World Journal

ISSN: 2356-6140
EISSN: 1537-744X
Publisher: Wiley

ARTICLE INFORMATION
==============================
PMCID: PMC6009717
PMID: 29973790
DOI: 10.1100/tsw.2002.16
Article ID: 684678
Article version: 1
Subjects: Short Report

Structural Proteomics of Eukaryotic Gene Families

Montelione Gaetano T. *
Anderson Stephen
Acton Thomas
Dixon Bonnie
Huang Yuanpeng
Moseley Hunter
Monleon Daniel
Shukla Kamal
Swapna Gaetano G. V. T.
Tejero Roberto
Center for Advanced Biotechnology Medicine, Rutgers University, Piscataway, NJ 08854, USA
*Gaetano T. Montelione: guy@cabm.rutgers.edu
Publication date: 2002
Electronic publication date: 2002 Jan 12
Volume: 2
First page: 32
Last page: 32
Copyright: Copyright © 2002 Gaetano T. Montelione et al.
Copyright year: 2002
License: This is an open access article distributed under the Creative Commons Attribution License, which permits unrestricted use, distribution, and reproduction in any medium, provided the original work is properly cited.
License URL: https://creativecommons.org/licenses/by/3.0/

==============================
Advances in Gene Technology: The Genome and Beyond –
Structural Biology for Medicine (Proceedings of the 2002 Miami
*Nature Biotechnology* Winter Symposium)
*TheScientificWorld* 2002, 2(S2), 32
ISSN 1532-2246; DOI 10.1100/tsw.2002.16

### STRUCTURAL PROTEOMICS OF EUKARYOTIC GENE FAMILIES

Gaetano T. Montelione*, Stephen Anderson, Thomas Acton, Bonnie Dixon, Yuanpeng Huang, Hunter Moseley, Daniel Monleon, Kamal Shukla, G.V.T. Swapna, and Roberto Tejero
Center for Advanced Biotechnology Medicine, Rutgers University, Piscataway, NJ 08854
*guy@cabm.rutgers.edu

**INTRODUCTION**. Genome sequencing projects have already determined nearly complete genome sequences of several organisms, including human. The products of these genes are widely recognized as the next generation of therapeutics and targets for the development of pharmaceuticals. While identification of these genes is proceeding quickly, elucidation of their three-dimensional (3D) structures and biochemical functions lags far behind. In some cases, knowledge of 3D structures of proteins can provide important insights into evolutionary relationships that are not easily recognized by sequence alignment comparisons. Thus, structure determination by NMR or X-ray crystallography can sometimes provide key information regarding protein fold class, locations and clustering of conserved residues, and surface electrostatic field distributions that connect a protein sequence with potential biochemical functions. The resulting limited set of putative biochemical functions can then be tested by appropriate biochemical assays.

**RESULTS**. We are developing technologies that significantly accelerate the process of protein structure determination and functional annotation by X-ray crystallography and NMR. These include bioinformatics methods for parsing novel genes into domain encoding regions, high-level "multiplexed" protein expression systems, database structures for keeping track of reagents and project data, and NMR pulse sequences, data collection methods, and expert-system software for automated analysis of protein resonance assignments and 3D structures. Examples will be presented of protein structures determined by the Northeast Structural Genomics Consortium (NESGC), a pilot project involving several institutes and utilizing both X-ray crystallography and NMR spectroscopy for structure and function analysis. The NESGC focuses on protein families that include representatives coded by genomes of metazoan organisms. These structures include several examples of novel folds, as well as protein structures that provide new insights into biochemical functions. The goal of this work is to develop a "high-throughput" process for structural analysis of proteins on a genomic scale and to apply this in the analysis of protein families identified in the genome sequencing projects.

### REFERENCES

1. Montelione, G.T. and Anderson, S. (1999) Nat. Struct. Biol. 6, 11–12.
2. Moseley, H.N.B. and Montelione, G.T. (1999) Curr. Opin. Struct. Biol. 9, 635–641.
3. Montelione, G.T., Zheng, D., Huang, Y.J., Gunsalus, K.C., and Szyperski, T.

(2000) Nat. Struct. Biol. 7, 982–986.
